# Accurate diagnosis of colorectal cancer based on histopathology images using artificial intelligence

**DOI:** 10.1186/s12916-021-01942-5

**Published:** 2021-03-23

**Authors:** K. S. Wang, G. Yu, C. Xu, X. H. Meng, J. Zhou, C. Zheng, Z. Deng, L. Shang, R. Liu, S. Su, X. Zhou, Q. Li, J. Li, J. Wang, K. Ma, J. Qi, Z. Hu, P. Tang, J. Deng, X. Qiu, B. Y. Li, W. D. Shen, R. P. Quan, J. T. Yang, L. Y. Huang, Y. Xiao, Z. C. Yang, Z. Li, S. C. Wang, H. Ren, C. Liang, W. Guo, Y. Li, H. Xiao, Y. Gu, J. P. Yun, D. Huang, Z. Song, X. Fan, L. Chen, X. Yan, Z. Li, Z. C. Huang, J. Huang, J. Luttrell, C. Y. Zhang, W. Zhou, K. Zhang, C. Yi, C. Wu, H. Shen, Y. P. Wang, H. M. Xiao, H. W. Deng

**Affiliations:** 1 Department of Pathology, Xiangya Hospital, Central South University, Changsha, 410078 Hunan China; 2 Department of Pathology, School of Basic Medical Science, Central South University, Changsha, 410013 Hunan China; 3 Department of Biomedical Engineering, School of Basic Medical Science, Central South University, Changsha, 410013 Hunan China; 4 Department of Biostatistics and Epidemiology, The University of Oklahoma Health Sciences Center, Oklahoma City, OK 73104 USA; 5 Laboratory of Molecular and Statistical Genetics, College of Life Sciences, Hunan Normal University, Changsha, 410081 Hunan China; 6 Department of Deming Department of Medicine, Tulane Center of Biomedical Informatics and Genomics, Tulane University School of Medicine, 1440 Canal Street, Suite 1610, New Orleans, LA 70112 USA; 7 Centers of System Biology, Data Information and Reproductive Health, School of Basic Medical Science, School of Basic Medical Science, Central South University, Changsha, 410008 Hunan China; 8 Department of Pharmacology, Xiangya School of Pharmaceutical Sciences, Central South University, Changsha, 410078 Hunan China; 9 School of Life Sciences, Central South University, Changsha, 410013 Hunan China; 10 College of Information Science and Engineering, Hunan Normal University, Changsha, 410081 Hunan China; 11 Department of Pathology, Gongli Hospital, Second Military Medical University, Shanghai, 200135 China; 12 Department of Pathology, the Peace Hospital Affiliated to Changzhi Medical College, Changzhi, 046000 China; 13 Pathological Laboratory of Adicon Medical Laboratory Co., Ltd, Hangzhou, 310023 Zhejiang China; 14 Department of Pathology, First Affiliated Hospital of Hunan Normal University, The People’s Hospital of Hunan Province, Changsha, 410005 Hunan China; 15 Department of Pathology, the Third Xiangya Hospital, Central South University, Changsha, 410013 Hunan China; 16 Department of Pathology, Sun Yat-Sen University Cancer Center, Guangzhou, 510060 China; 17 Department of Pathology, Fudan University Shanghai Cancer Center, Shanghai, 200032 China; 18 Department of Pathology, Chinese PLA General Hospital, Beijing, 100853 China; 19 Department of Pathology, Nanjing Drum Tower Hospital, the Affiliated Hospital of Nanjing University Medical School, Nanjing, 210008 China; 20 Department of Pathology, The first affiliated hospital, Air Force Medical University, Xi’an, 710032 China; 21 Institute of Pathology and southwest cancer center, Southwest Hospital, Third Military Medical University, Chongqing, 400038 China; 22 Department of Pathology, Guangdong Provincial People’s Hospital, Guangdong Academy of Medical Sciences, Guangzhou, 510080 China; 23 Department of Anatomy and Neurobiology, School of Basic Medical Science, Central South University, Changsha, 410013 Hunan China; 24 School of Computing Sciences and Computer Engineering, University of Southern Mississippi, Hattiesburg, MS 39406 USA; 25 College of Computing, Michigan Technological University, Houghton, MI 49931 USA; 26 Department of Computer Science, Bioinformatics Facility of Xavier NIH RCMI Cancer Research Center, Xavier University of Louisiana, New Orleans, LA 70125 USA; 27 Department of Pathology, Ochsner Medical Center, New Orleans, LA 70121 USA; 28 Department of Statistics, Florida State University, Tallahassee, FL 32306 USA; 29 Division of Biomedical Informatics and Genomics, Deming Department of Medicine, Tulane University School of Medicine, New Orleans, LA 70112 USA; 30 Department of Biomedical Engineering, Tulane University, New Orleans, LA 70118 USA

**Keywords:** Colorectal cancer, Histopathology image, Deep learning, Cancer diagnosis

## Abstract

**Background:**

Accurate and robust pathological image analysis for colorectal cancer (CRC) diagnosis is time-consuming and knowledge-intensive, but is essential for CRC patients’ treatment. The current heavy workload of pathologists in clinics/hospitals may easily lead to unconscious misdiagnosis of CRC based on daily image analyses.

**Methods:**

Based on a state-of-the-art transfer-learned deep convolutional neural network in artificial intelligence (AI), we proposed a novel patch aggregation strategy for clinic CRC diagnosis using weakly labeled pathological whole-slide image (WSI) patches. This approach was trained and validated using an unprecedented and enormously large number of 170,099 patches, > 14,680 WSIs, from > 9631 subjects that covered diverse and representative clinical cases from multi-independent-sources across China, the USA, and Germany.

**Results:**

Our innovative AI tool consistently and nearly perfectly agreed with (average Kappa statistic 0.896) and even often better than most of the experienced expert pathologists when tested in diagnosing CRC WSIs from multicenters. The average area under the receiver operating characteristics curve (AUC) of AI was greater than that of the pathologists (0.988 vs 0.970) and achieved the best performance among the application of other AI methods to CRC diagnosis. Our AI-generated heatmap highlights the image regions of cancer tissue/cells.

**Conclusions:**

This first-ever generalizable AI system can handle large amounts of WSIs consistently and robustly without potential bias due to fatigue commonly experienced by clinical pathologists. It will drastically alleviate the heavy clinical burden of daily pathology diagnosis and improve the treatment for CRC patients. This tool is generalizable to other cancer diagnosis based on image recognition.

**Supplementary Information:**

The online version contains supplementary material available at 10.1186/s12916-021-01942-5.

## Background

Colorectal cancer (CRC) is the third leading cancer by incidence (6.1%) but second for mortality (9.2%) worldwide [1]. The global burden of CRC is expected to increase 60% by 2030, in terms of new cases and deaths [2]. The accurate and prompt detection of CRC is essential to improve treatment effectiveness and survivorship. The current diagnosis of CRC requires an extensive visual examination by highly specialized pathologists. Diagnoses are made using digital whole-slide images (WSIs) of the hematoxylin and eosin (H&E)-stained specimens obtained from formalin-fixed paraffin-embedded (FFPE) or frozen tissues. The challenges for the WSI analysis include very large image size (> 10,000 × 10,000 pixels), histological variations in size, shape, texture, and staining of nuclei, making the diagnosis complicated and time-consuming [3]. In most modern pathology departments, the average consultative workload increases by ~ 5–10% annually [4]. The current trends indicate a shortage of pathologists around the world, including USA [5] and low- to middle-income countries [6]. This results in overworked pathologists, which can lead to higher chances of deficiencies in their routine work and dysfunctions of the pathology laboratories with more laboratory errors [4]. While the demands of colon specimen examination in gastroenterology clinics are high, the training time of pathologists is long (> 10 years) [7]. It is thus imperative to develop reliable tools for pathological image analysis and CRC detection that can improve clinical efficiency and efficacy without unintended human bias during diagnosis.

State-of-the-art artificial intelligence (AI) approaches, such as deep learning (DL), are very powerful in classification and prediction. There have been many successful applications of DL, specifically convolutional neural network (CNN), in WSI analysis for lung [8, 9], breast [10, 11], prostate [12–14], and skin [15, 16] cancers. Most of the existing CNN for the CRC WSI analysis focused on the pathology work after cancer determination, including grade classification [17], tumor cell detection and classification [18–20], and survivorship prediction [21–23]. Although they resulted in reasonably high accuracy, their study sample sizes are limited and do not fully represent the numerous histologic variants of CRC that have been defined. These variants include tubular, mucinous, signet ring cell, and others [24]. These limitations inflate prediction error when applied to different independent samples. Meanwhile, most of the current DL models were developed from single data source without thorough validation using independent data. They only calculated the accuracy of patches without diagnosing WSIs or the patients. Their general applicability for CRC WSI diagnosis in various clinical settings, which may involve heterogeneous platforms and image properties, remains unclear. A DL approach generalizable to daily pathological CRC diagnosis that relieves clinical burden of pathologists and improves diagnostic accuracy is yet to be developed [25].

Here, we developed a novel automated AI approach centered on weakly labeled supervised DL for the very first general clinical application of CRC diagnosis. This AI approach uses Inception-v3 CNN architecture [26] with weights initialized from transfer learning. Weakly labeled supervised learning is advantageous in training massive and diverse datasets without exact labelling at object levels (e.g., small cancer cells) [12]. Transfer learning is a highly effective and efficient DL technique for image analysis that can utilize previously learned knowledge on general images for medical image analyses [27]. Our work is based on WSIs from multiple independent hospitals/sources in China (8554 patients), USA (1077 patients), and Germany (> 111 slides). This study has high practical value for improving the effectiveness and efficiency of CRC diagnosis and thus treatment. It highlights the general significance and utility of the application of AI to image analyses of other types of cancers.

## Methods

### Colorectal cancer whole-slide image dataset

We collected 14,234 CRC WSIs from fourteen independent sources (Table 1). All data were de-identified. The largest image set was from 6876 patients admitted between 2010 and 2018 in Xiangya Hospital (XH), Central South University (CSU, Changsha, China). XH is the largest hospital in Hunan Province and was established in 1906 with a close affiliation with Yale University [28]. The other independent sources were The Cancer Genome Atlas (TCGA) of the USA (https://portal.gdc.cancer.gov/) [29], the National Centre for Tumor Diseases (NCT) biobank and the University Medical Center Mannheim (UMM) pathology archive (NCT-UMM) of Germany (https://zenodo.org/record/1214456#.XgaR00dTm00, [22]), Adicon Clinical Laboratories (ACL), INC, and eleven hospitals in China (detailed in Table 1). The hospitals involved are located in the major metropolitan areas of China serving > 139 million population, including those most prestigious hospitals in pathology in China: XH, Fudan University Shanghai Cancer Center (FUS), Chinese PLA General Hospital (CGH), Southwest Hospital (SWH), and The First Affiliated Hospital Air Force Medical University (AMU); other state-level esteemed hospitals: Sun Yat-Sen University Cancer Center (SYU), Nanjing Drum Tower Hospital (NJD), Guangdong Provincial People’s Hospital (GPH), Hunan Provincial People’s Hospital (HPH), and The Third Xiangya Hospital of CSU (TXH); and a regional reputable Pingkuang Collaborative Hospital (PCH). All WSIs were from FFPE tissues, except parts (~ 75%) of TCGA WSIs were from frozen tissues [30]. The process of collection, quality control, and digitalization of the WSIs is described in Supplementary-Text 1.a (see Additional file 1).

**Table 1:** Usage of datasets from multicenter data source

Data source	Dataset usage	Sample preparation	Examination type Radical surgery/colonoscopy	Population^*^	CRC		Non-CRC		Total	
					Subjects	Slides	Subjects	Slides	Subjects	Slides
Xiangya Hospital (XH)	A	FFPE	100% / 0%	Changsha, China	614	614	228	228	842	842
NCT-UMM (NCT-CRC-HE-100 K)	B	FFPE	NA	Germany	NA	NA	NA	NA	NA	86
NCT-UMM (CRC-VAL-HE-7 K)	B	FFPE	NA	Germany	NA	NA	NA	NA	NA	25
XH	C	FFPE	80% / 20%	Changsha, China	3990	7871	1849	2132	5839	10,003
XH	D	FFPE	89% / 11%	Changsha, China	98	99	97	114	195	213
Pingkuang Collaborative Hospital (PCH)	C & D	FFPE	60% / 40%	Jiangxi, China	50	50	46	46	96	96
The Third Xiangya Hospital of CSU (TXH)	C & D	FFPE	61% / 39%	Changsha, China	48	70	48	65	96	135
Hunan Provincial People’s Hospital (HPH)	C & D	FFPE	61% / 39%	Changsha, China	49	50	49	49	98	99
ACL	C & D	FFPE	22% / 78%	Changsha, China	100	100	107	107	207	207
Fudan University Shanghai Cancer Center (FUS)	C & D	FFPE	97% / 3%	Shanghai, China	100	100	98	98	198	198
Guangdong Provincial People’s Hospital (GPH)	C & D	FFPE	77% / 23%	Guangzhou, China	100	100	85	85	185	185
Nanjing Drum Tower Hospital (NJD)	C & D	FFPE	96% / 4%	Nanjing, China	100	100	97	97	197	197
Southwest Hospital (SWH)	C & D	FFPE	93% / 7%	Chongqing, China	99	99	100	100	199	199
The First Affiliated Hospital Air Force Medical University (AMU)	C & D	FFPE	95% / 5%	Xi’an, China	101	101	104	104	205	205
Sun Yat-Sen University Cancer Center (SYU)	C & D	FFPE	100% / 0%	Guangzhou, China	91	91	6	6	97	97
Chinese PLA General Hospital (CGH)	C	FFPE	NA	Beijing, China	0	0	100	100	100	100
TCGA (TCGA-Frozen)	C	Frozen	100% / 0%	U.S.	631	1214	110	133	631^**^	1347
TCGA (TCGA-FFPE)	C	FFPE	100% / 0%	U.S.	441	441	5	5	446	446
**Total**					6612	11,100	3129	3469	9631	14,680

^*^Location map available in Supplementary Text 1.a (see Additional file 1). ^**^For the TCGA –Frozen data only, the non-CRC slides were made with normal intestinal tissues on part of the CRC slides

**Fig. 1:**
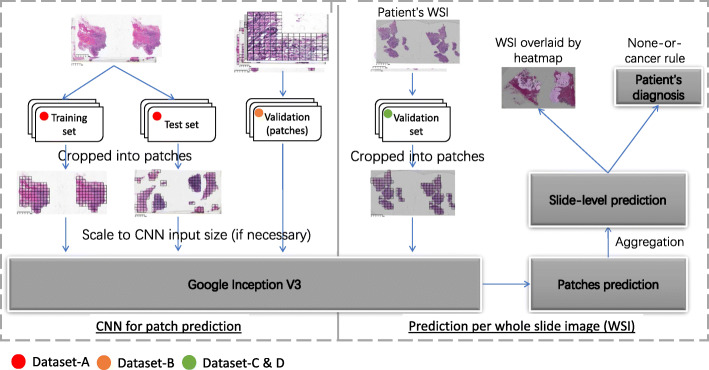
Study pipeline and dataset usage

We formed four datasets (Table 1). Dataset-A includes slides from only XH and was used for patch-level training and testing (Table 2). We carefully selected WSIs to include all common tumor histological subtypes. Using incomplete information of cancer cells/tissues (e.g., location, shape, and demarcation), pathologists weakly labeled the patches from WSIs as either containing or not cancer cells/tissues. Two weakly labeled patches were provided as illustrative comparative examples with two fully labeled patches serving as contrasts (see Additional file 1: Supplementary-Figure 1). Patches from the same patient were all put into the same data set (either training or testing) so that the training and testing data sets are independent. To ensure an appropriate and comprehensive representation of cancer and normal tissue characteristics, we included an average of 49 patches per tumor sample and 144 patches per healthy sample. The number of patches containing a large proportion of cancer cells and the number of patches containing only a few cancer cells were approximately balanced so that the patches used for training were representative of cases seen in practice.

**Table 2:** Dataset-A (training and testing) and Dataset-B (external validation) for patch-level analysis

Dataset	Cancer			Non-cancer			Total		
	Subjects	Slides	Patches	Subjects	Slides	Patches	Subjects	Slides	Patches
**Training**	406	406	19,940	153	153	22,715	559	559	42,655
**Testing**	208	208	10,116	75	75	10,148	283	283	20,264
**Validation**	NA	NA	15,550	NA	NA	91,630	NA	111	107,180^*^
**Total**	> 614	> 614	45,606	> 228	> 228	124,493	> 842	953	170,099

* There are two datasets used for validation. The number is the sum of the two datasets

Patch-level performance was further validated using Dataset-B, which contained 107,180 patches downloaded from NCT-UMM. There were two independent subsets: 100,000 image patches of 86 hematoxylin and eosin stain (HE) slides of human cancer tissue (NCT-CRC-HE-100K) and 7180 image patches of 25 slides of CRC tissue (CRC-VAL-HE-7K) [22]. The overall split for patch-level training, testing, and external validation was about 2:1:5. All images are 224 × 224 pixels at 0.5 μm per pixel. More description can be found at https://zenodo.org/record/1214456#.XV2cJeg3lhF. The patches were rescaled to default input size before they are fed to the networks for testing.

**Fig. 2:**
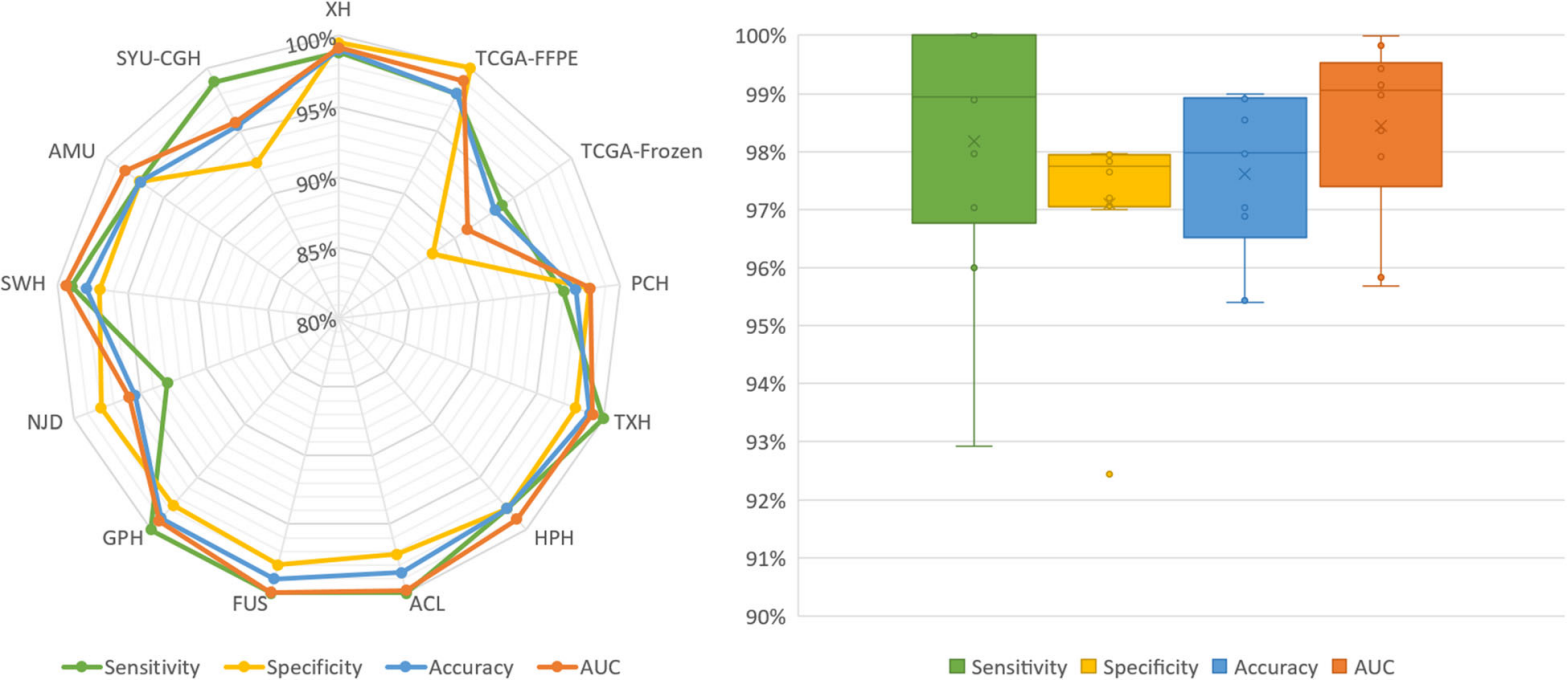
Patient-level testing performance on twelve independent datasets from Dataset-C. Left: the radar map of the sensitivity, specificity, accuracy, and AUC in each dataset from Dataset-C. Right: the boxplot showing the distribution of sensitivity, specificity, accuracy, and AUC in datasets excluding XH and TCGA. The horizontal bar in the box indicates the median, while the cross indicates the mean. Circles represent data points

Dataset-C was used for patient-level validation and is composed of slides from XH, the other hospitals, ACL, and frozen and FFPE samples of TCGA. Given the high imbalance of cancer and non-cancer slides in SYU and CGH (Table 1), they were combined in Dataset-C. In Dataset-C, the area occupied by cancer cells varied in images from different centers. Most (~ 72%) of the slides from the ten hospitals and ACL contained 10–50% cancer cells by area (see Additional file 1: Supplementary-Figure 2).

Dataset-D was used for the Human-AI contest and contained approximately equal number of slides from XH, the other hospitals, and ACL. There is an average of ~ 5045 patches on each slide, and more than 20% of the slides contain < 1000 patches. Supplementary-Text 1.b summarized the allocation of slides in the different datasets (see Additional file 1).

After the slides were digitalized, the visual verification of the cancer diagnosis labels was performed with high stringency and accuracy. Dataset-A and Dataset-C included more than 10,000 slides, which were independently reviewed by two senior and seasoned pathologists with initial and second read. When their diagnoses were consistent with the previous clinical diagnosis conclusion, the slides were then included in the dataset. If the two experts disagreed with each other or with the previous clinical diagnosis, the slides were excluded. The labels of slides from TCGA were obtained from the original TCGA database. The labels of Dataset-B were from the NCT-UMM. The binary labels of Dataset-D for the Human-AI contest were more strictly checked. Three highly experienced senior pathologists independently reviewed the pathological images without knowing the previous clinical diagnosis. If a consensus was reached, the slides were included; otherwise, two other independent pathologists would join the review. After a discussion among the five pathologists, the sample was included only if they reached an agreement; otherwise, it was excluded.

### Study design and pipeline

Our approach to predict patient cancerous status involved two major steps: DL prediction for local patches and patch-level results aggregation for patient-level diagnosis (Fig. 1). The WSIs after preprocessing served as the input for patch-level prediction. A deep-learning model was constructed to analyze the patches. The patch-level prediction was then aggregated by a novel patch-cluster-based approach to provide slide and patient-level diagnosis. The performance of patch-level prediction and the way of aggregation would determine to a large extent the accuracy of patient-level diagnosis. Our empirical results showed that a patch-level sensitivity of ~ 95% and specificity of ~ 99% was sufficient to achieve a high predictive power and control the false positive rate (FPR) at the patient-level using our proposed aggregation approach (see Additional file 1: Supplementary-Text 1.c). In addition, the heatmap and activation map were generated to show the informative area on the slide. The details for each step are illustrated as follows.

### Image preprocessing for patch-level training

There were 3 steps in the image preprocessing. First, we tiled each WSI at × 20 magnification with non-overlapping 300 × 300 pixel patches, which can be easily transformed to the required input size of most CNN architectures (such as the 299 × 299 input size required by Inception-v3 [26], see Additional file 1: Supplementary-Table 1). The use of a smaller patch size compared with other studies with patches of 512 × 512 pixels would make the boundaries of cancer regions more accurate [19]. Second, we removed non-informative background patches according to two criteria: the maximum difference among the 3 color channel values of the patch was less than 20, or the brightness of more than 50% of the patch surface was less than 220 in grayscale [8]. Combining these two criteria, we removed background patches and kept as many tissue patches as possible. Third, regular image augmentation procedures were applied, such as random flipping and random adjustment of the saturation, brightness, contrast, and hue. The color of each pixel was centered by the mean of each image and its range was converted/normalized from [0, 255] to [− 1, 1].

### Patch-level training by deep learning

Our DL model used Inception-v3 as the CNN architecture to classify cancerous and normal patches. The Inception network uses different kernel sizes and is specifically powerful in learning diagnostic information in pathological image from differing scales. This architecture has achieved near human expert performance in the analyses of other cancer types [8, 15, 31, 32]. There are a few Inception architectures performed well on the ImageNet dataset [33] and WSIs analysis [33], such as the Inception-v1 [34], Inception-v3 [26], and Inception-v4 [35]. We chose Inception-v3 based on extensive comparison of their patch-level and patient-level performance in testing sets, which showed that the complexity and multiscale modules in Inception-v3 made it more appropriate to recognize the histopathology WSIs (see Additional file 1: Supplementary-Text 1.d) [26, 34–39]. During the study, we also tested some most recent algorithms, such as DenseNet [37] and ResNeXt [39]. Inception-v3 still performs best at the patch-level CRC classification.

We initialized the CNN by transfer learning with pre-trained weights from ImageNet [26], which were optimized to capture the structures in general images [27]. With transfer learning, our model can recognize pivotal image features for CRC diagnosis most efficiently. The 300 × 300 pixel patches were resized to a size of 299 × 299 pixels. Accordingly, the patches in the testing sets were rescaled to 299 × 299 pixels (0.37 μm/pixels) before they were fed to the network. The network was deeply fine-tuned by following training steps. Given the possible high false positive rate after aggregating the patch-level results, the optimal set of hyper-parameters was randomly searched with an objective of reaching > 95% sensitivity and > 99% specificity. We showed that, with this objective at the patch level, the error rate at the patient level was well controlled (see Additional file 1: Supplementary-Text 1.c). The network was finalized after 150,000 epochs of fine-tuning the parameters at all layers using the RMSProp [40] optimizer with a weight decay of 0.00004, a momentum value of 0.9, and RMSProp decay set to 0.9. The initial learning rate was 0.01 and was exponentially decayed with epochs to the final learning rate of 0.0001. The optimized result was achieved when the batch size was 64. The training and testing procedures were implemented in a Linux server with an NVIDIA P100 GPU. We used Python v2.7.15 and Tensorflow v1.8.0 for data preprocessing and CNN model training and testing.

### Patient diagnosis and false positive control

Considering the high false positive rate (FPR) accumulated from multiple patch-level predictions, we proposed a novel patch-cluster-based aggregation method for slide-level prediction based on the fact that the tumor cells tend to gather together (especially at × 20 magnification). Motivated by the clustering inference of fMRI [41], we predicted the WSI as cancer positive if there were several positive patches topologically connected as a cluster on the slide (defined by the cluster size), such as four patches as a square. Otherwise, we predicted the slide as negative. We tested various cluster sizes and chose a cluster size of four as the result of an empirically observed best balance of sensitivity and FPR in the testing dataset (see Additional file 1: Supplementary-Text 1.e). For a patient who had one or multiple slides, denoted by *S* = {*s*_1_, *s*_2_, …, *s*_*l*_}, we provided the patient-level diagnosis *D*(*S*) combining the results from all of the patient’s slides: *D*(*S*) = *D*(*s*_1_) ∪ *D*(*s*_2_) ∪ … ∪ *D*(*s*_*l*_), where *D*(*s*_*l*_)= 1 or 0 indicated a positive or negative classification of the *l*th slide respectively. The patient will be diagnosed as having cancer as long as one of the slides indicates diagnosis.

### Human-AI contest

Six pathologists (A-F) with varying experience of 1 to 18 clinical practice years joined the contest (see Additional file 1: Supplementary-Table 2). The pathologists independently provided a diagnosis specifying cancer or non-cancer for each patient after reading the WSIs in Dataset-D. The pathologists did not participate in the data collection or labeling. An independent analyst blindly summarized and compared the accuracy and speed of AI and human experts in performing diagnosis.

### Statistical analysis and visualization

We assessed the performance of the AI and pathologists in terms of sensitivity, specificity, and accuracy ($\frac{\#\mathrm{of}\ \mathrm{correct}\ \mathrm{predictions}}{\#\mathrm{of}\ \mathrm{total}\ \mathrm{predictions}}$) for the diagnosis. The receiver operating characteristic (ROC) curve that plotted the sensitivity versus the FPR and the corresponding area under the ROC curve (AUC) were computed. The AUCs of AI and each of the pathologists in multiple datasets were compared by the paired Wilcoxon signed-rank test. We examined the pairwise agreements among AI and pathologists by Cohen’s Kappa statistic (*K*). The statistical analyses were done in R v3.5 (Vienna, Austria), using packages caret, ggplot2, pROC, and psych among others. Statistical significance level was set at an alpha level of 0.05.

To locate the CRC region in the WSI, we visualized the WSI as a heatmap based on the confidence score of each patch. Brighter regions indicate higher confidence that the classifier would consider the region cancer positive. The heatmap was generated by Python (https://www.python.org/) and overlaid with the original WSI by gimp (https://www.gimp.org/).

## Results

### Highest accuracies in patch-level prediction by our model

We divided the 842 WSIs from Dataset-A (Table 1) into 62,919 non-overlapping patches (Table 2) to construct the CNN for patch-level prediction based on fine-tuning of Inception-v3. An average of ~ 75 patches per WSI were included to ensure an appropriate and comprehensive representation of cancer and normal tissue characteristics. Three major CRC histological subtypes were involved for the training and testing, including 74.76% tubular, 24.59% mucinous, and 0.65% signet ring cell patches, roughly reflecting their clinical incidences [42]. In the training, 19,940 (46.75%) patches had cancer, and 22,715 (53.25%) patches were normal. Using another independent set of 10,116 (49.92%) cancer and 10,148 (50.08%) non-cancer patches, the AI for patch-level prediction achieved a testing accuracy of 98.11% and an AUC of 99.83%. The AUC outperformed that of all the previous AI studies for CRC diagnosis and prediction (79.2–99.4%) and even for the majority of other types of cancer (82.9–99.9%, see Additional file 1: Supplementary-Tables 3, [8, 12, 17, 19, 22, 43–48]). The specificity was 99.22% and the sensitivity 96.99%, both outstanding. In the external validation Dataset-B, our model yielded an accuracy and AUC of 96.07% and 98.32% in NCT-CRC-HE-100 K, and 94.76% and 98.45% in CRC-VAL-HE-7 K, which matched the performance from in-house data and outplayed the patch-level validation analysis in other AI studies (AUC 69.3–95.0%, see Additional file 1: Supplementary-Table 3). The patch-level testing and validation result was summarized in Table 3.

**Table 3:** Patch-level (Dataset-A and Dataset-B) and patient-level (Dataset-C and Dataset-D) performance summary

Source	Sensitivity	Specificity	Accuracy	AUC
**Dataset-A (patch-level testing)**				
XH	96.99%	99.22%	98.11%	99.83%
**Dataset-B (patch-level validation)**				
NCT-CRC-HE-100 K	92.03%	96.74%	96.07%	98.32%
CRC-VAL-HE-7 K	94.24%	94.87%	94.76%	98.45%
**Dataset-C (patient-level validation)**				
XH	98.80%	99.51%	99.02%	99.16%
TCGA-Frozen	94.04%	88.06%	93.44%	91.05%
TCGA-FFPE	97.96%	100.00%	97.98%	98.98%
SYU-CGH	98.90%	92.45%	95.43%	95.68%
**Dataset-D (patient-level Human-AI contest)**				
XH	97.96%	100%	98.97%	98.99%
SYU	98.90%	100%	98.97%	99.45%
**Dataset-C and Dataset-D (patient-level validation and Human-AI contest)**				
PCH	96.00%	97.83%	96.88%	97.91%
TXH	100%	97.92%	98.96%	99.20%
HPH	97.96%	97.96%	97.96%	98.98%
FUS	100%	97.96%	98.99%	99.99%
GPH	100%	97.65%	98.91%	99.15%
NJD	92.93%	97.94%	95.41%	95.84%
SWH	98.99%	97.00%	97.99%	99.42%
AMU	97%	97.06%	97.04%	98.37%
ACL	100%	97.20%	98.55%	99.83%

### Diagnosis of CRC at patient level using DL-predicted patches

Our AI approach was tested for patient diagnosis with 13,514 slides from 8594 patients (Dataset-C). In the largest subset (5839 patients) from XH, our approach produced an accuracy of 99.02% and an AUC of 99.16% (Fig. 2, Table 3). In other independent multicenter datasets, our approach consistently performed very well. For the FFPE slides from other hospitals, TCGA-FFPE, and ACL, the AI approach yielded an average AUC and accuracy higher than 97.65% (Fig. 2). For frozen slides TCGA-Frozen, the AI accuracy and AUC were 93.44% and 91.05% respectively (Fig. 2). Our AUC values (ranging from 91.05 to 99.16%) were higher than that of other AI-based approaches for independent datasets (ranging from 83.3 to 94.1%). Of note, because the majority of those earlier AI approaches were tested on datasets of much smaller sample sizes (see Additional file 1: Supplementary-Table 3), their performances may be over-estimated. The limited number of negative slides in TCGA may result in an imbalanced classification problem that needs further investigation, which is beyond the scope of this study. The results on TCGA-Frozen slides showed that our method did learn the histological morphology of cancer and normal tissues for cancer diagnosis, which is preserved in both the FFPE and frozen samples, even though our method was developed based on the FFPE samples. Table 3 summarized the complete patient-level result.

### Contest with six human experts

**Fig. 3:**
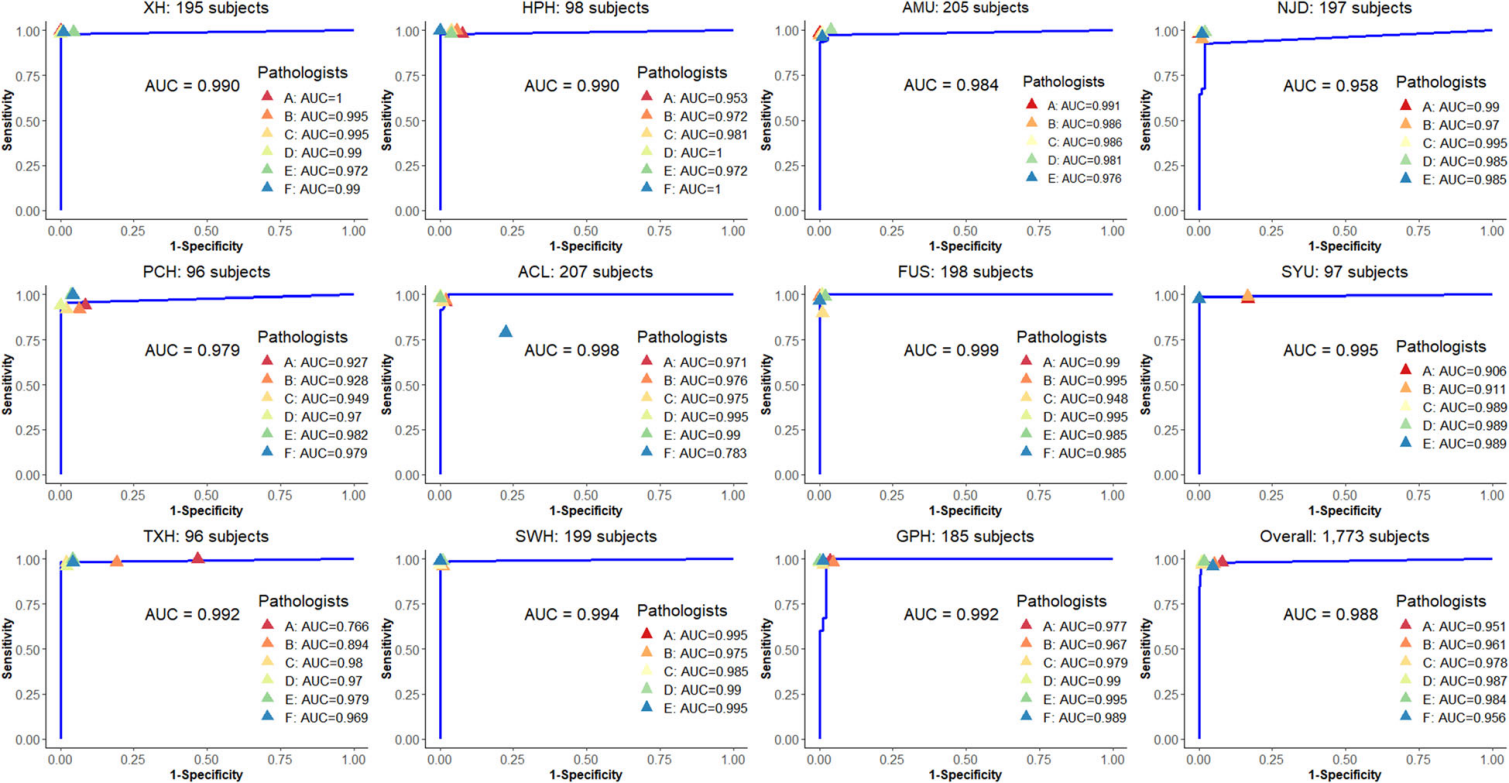
ROC analysis of AI and pathologists in the Human-AI contest using Dataset-D. The blue line is the estimated ROC curve for AI. The colored triangles indicate the sensitivity and specificity achieved by the six pathologists

The performance of our AI approach was consistently comparable to the pathologists in diagnosing 1831 WSIs from independent centers (Dataset-D, Fig. 3). The AI resulted in an average accuracy and AUC of 98.06% (95% confidence interval [CI] 97.36 to 98.75%) and 98.83% (95% CI 98.15 to 99.51%), which both ranked top three out of the seven competitors (AI plus the six pathologists) and were greater than the average of the pathologists (accuracy 97.14% (95% CI 96.12 to 98.15%) and AUC 96.95% (95% CI 95.74 to 98.16%)). The paired Wilcoxon signed-rank test of AUCs in multicenter datasets found there were no significant differences between AI and each of the pathologists. The AI yielded the highest sensitivity (98.16%) relative to the average (97.47%) of the pathologists (see Additional file 1: Supplementary-Table 4). The pathologists (D and E) who slightly outperformed the AI have 7 and 12 years of clinical experience respectively, while the AI outperformed the other 4 pathologists with 1, 3, 5, and 18 years of experience respectively. Cohen’s Kappa statistic (*K*) showed an excellent agreement (*K* ≥ 0.858, average 0.896) between AI and every pathologist (see Additional file 1: Supplementary-Table 5). Our approach is thus proven generalizable to provide diagnosis support for potential CRC subjects like an independent pathologist, which can drastically relieve the heavy clinical burden and training cost of professional pathologists. Details of the Human-AI contest are given in Supplementary-Tables 4 & 5 (see Additional file 1)

The pathologists were all informed to compete with our AI and with each other; hence, their performances were achieved under their best possible conditions with very best effort, which represented their highest skill with least error. However, with heavy workload in clinic, their performance in terms of accuracy and speed will not be as stable as that of AI. The current study of AI in cancer diagnosis using WSI has shown that AI can accurately diagnose in ~ 20 s [8] or less (~ 13 s in our case). With evolved DL techniques and advanced computing hardware, the AI can constantly improve and provide steady, swift, and accurate first diagnosis for CRC or other cancers.

### Slide-level heatmap

**Fig. 4:**
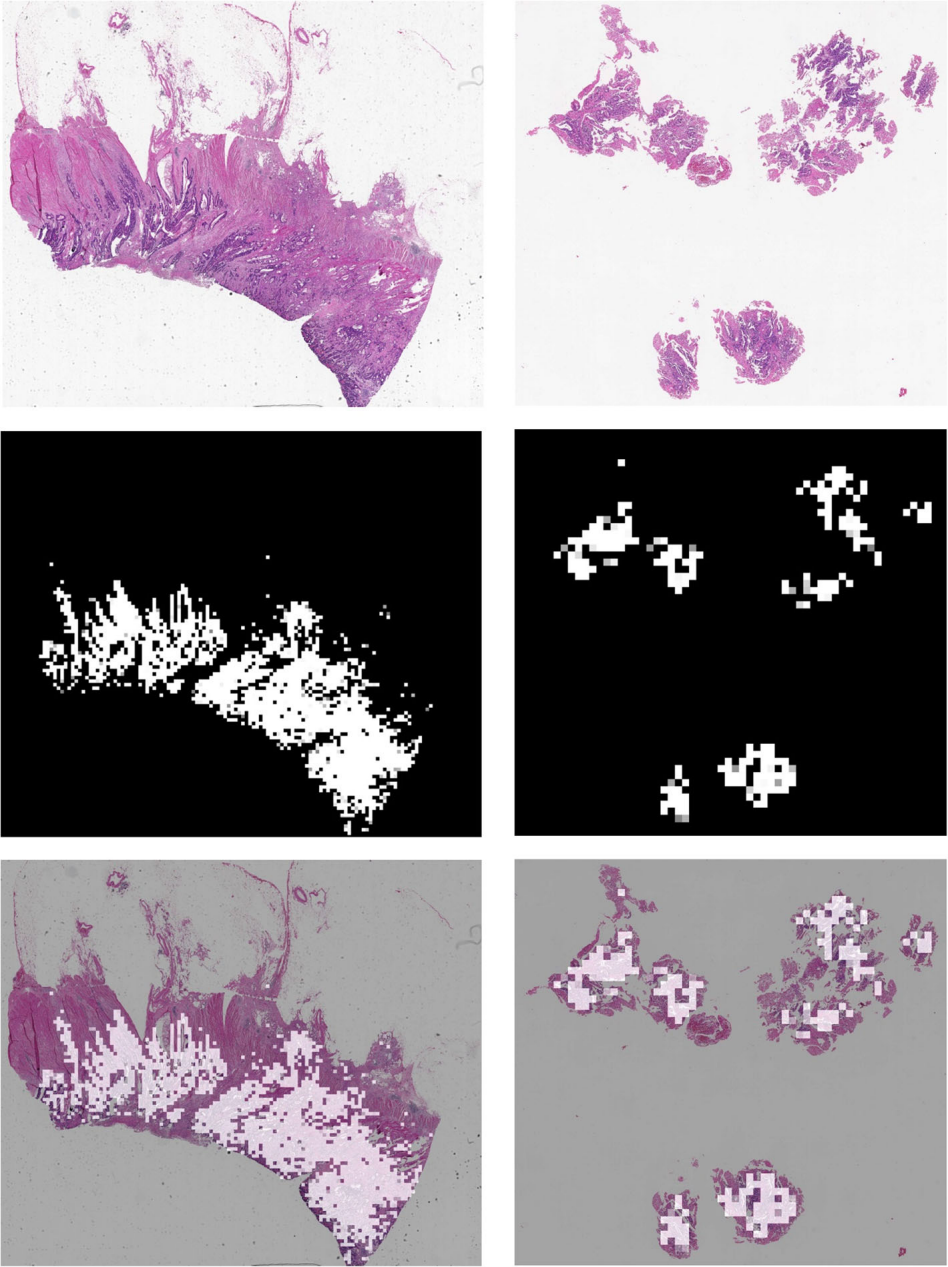
Heatmap produced by AI. Top row: WSI from radical surgery (left) and colonoscopy (right); middle row: AI predicted heatmap corresponding to the first row, with white coloration indicating predicted cancer region; bottom row: heatmap overlaid on the corresponding WSI

Our approach offers an additional distinct feature: heatmap for highlighting potential cancer regions (as patches) in WSI. In Fig. 4, we presented two WSIs, which were overlaid with the predicted heatmap. For both radical surgery WSI and colonoscopy WSI, the true cancerous region was highly overlapped with highlighted patches obtained by AI, which was also verified by pathologists. See more examples in Supplementary-Figure 3 (see Additional file 1). In addition, to visualize informative regions utilized by DL for the CRC detection, we provided the activation maps in Supplementary-Figure 4 (see Additional file 1).

## Discussion

We collected high-quality, comprehensive, and multiple independent human WSI datasets for training, testing, and external validation of our AI-based approach focusing on pathological diagnosis of CRC under common clinical settings. We mimicked the clinical procedure of WSI analysis, including the image digitalization, slide review, and expert consultations of the disputed slides. Different from other studies [21], we did not apply any manual selection of slides or the area of interest when building the study dataset. Given the complex histologic variants of CRC, we randomly selected training patches from three most commonly seen subtypes roughly proportional to their incidences. The number of patches from images with large and small cancer tissue area was balanced and well represented in patch-level analysis. The collected images were labeled by agreement of at least two senior experts in CRC pathology (see Additional file 1: Supplementary-Text 1.b). The testing dataset from different locations in China, USA, and Germany served as a representative pool for validation and generalization. Our dataset well represents the slides seen in clinics. Consequently, the trained AI model is robust and generalizable to analyze images of different production protocols and image quality.

For a fast-growing area, we are aware of that several new CNN architectures have been proposed after the completion of the study of the present paper, such as the DenseNet [37], Squeeze-and-Excitation network [38], and ResNeXt [39]. We did some exploratory analysis by comparing the ResNet152V2, DenseNet201, and NASNetLarge relative to the Inception-v3 in classifying patches. DenseNet201 produced similar evaluation metrics as Inception-v3, while the other two architectures yielded less accuracy and AUC than Inception-v3. Although these new models have been shown to increase the prediction accuracy on ImageNet dataset compared to Inception-v3, the complexity (depth and number of parameters) and the multiscale modules in Inception-v3 may be appropriate to recognize the CRC WSIs. The performance of the new architectures on pathology images analysis and cancer diagnosis deserves more focused dedicated research for more detailed technical comparison. Moreover, we identify other techniques that may extend the current study, such as the semi- and unsupervised learning [49, 50], which can learn from more WSIs with and without labels efficiently, and the multiscale decision aggregation and data augmentation [51], which can work in the presence of limited data. Given the highly accurate performance already achieved in the current approach presented, we can investigate if and how these new techniques might attain the current prediction performance with less data collection and labelling effort in future studies.

There are several histological types that were too rare (less than 0.5% in incidence [52]) to be included, such as medullary, micropapillary, and serrated. Our AI approach performed only slightly less satisfactory in frozen samples than in FFPE samples. With WSIs from rare types and more frozen samples available for training in the future, we expect our approach can be constantly improved to be more generalizable.

Most of the previous studies obtained the patient’s diagnosis by integrating the patch-level recognition results, since it is not feasible to process the large-size WSI directly. This strategy is difficult to control the accumulated false positive rate (FPR) from multiple predictions based on individual patches. Recently, Coudray et al. used the proportion of positive patches or the average probability of all patches as the prediction criterion for the WSI [8]. Although their results were verified in three independent datasets (all with small sample sizes (340 slides)), their aggregation method may not be valid for those images with only a small area of cancer tissues where it will yield false negative findings for cancer patients. Instead, we proposed a novel aggregation strategy for patch-based WSI or patient-level prediction, which is intuitive and can easily balance the sensitivity and specificity. Specifically, we aggregated information from the cluster of patches that are topologically connected on the slide to determine the cancer status. In practice, setting the cluster size to four is most likely to exceed the average accuracy of pathologists, while cluster size of two can be used for pathological screening with an average sensitivity of ~ 99.78% and an average specificity of ~ 72.29% according to our test data (see Additional file 1: Supplementary-Text 1.e).

## Conclusions

In summary, we developed a novel AI-based histopathological image classification approach for CRC diagnosis using deep learning, which achieved the best performance with the largest number of sample sizes and data sources in the field so far. Our approach was able to quickly and accurately distinguish CRC cases from healthy or inflammatory cases and was comparable to or even superior to pathologists in the testing of large-scale multicenter data. To the best of our knowledge, this is the first AI study for a reliable, generalized, and robust auxiliary tool for daily clinical pathology diagnosis of CRC initial screening. Our approach may also be adapted and applied to the histological analysis of other cancer types via the code available upon request.

## Supplementary Information

**Additional file 1:** **Supplementary-Text 1.a** Collection and digitalization of the WSIs. **Supplementary-Text 1.b** Dataset-A, B, C and D. **Supplementary-Text 1.c** Patch-level performance and patient-level accuracy. **Supplementary-Text 1.d** Comparison of different architectures at patch-level. **Supplementary-Text 1.e** Comparison of different cluster sizes for aggregation of patch-level results. **Supplementary-Table 1.** Input patch size for common CNN. **Supplementary-Table 2** Pathologist info. **Supplementary-Table 3** List of AUCs of AI applied in CRC and other cancer types. **Supplementary-Table 4** Overall performance of AI and pathologists in Human-AI contest. **Supplementary-Table 5** Cohen’s Kappa coefficient for agreement among human experts and AI. **Supplementary-Figure 1** Weakly-labeled and fully-labeled CRC patches. **Supplementary-Figure 2**.The distribution of cancerous area in multiple independent WSI datasets measured by the proportion of patches (P) containing cancer cells on the WSI. **Supplementary-Figure 3** Heatmap produced by AI. **Supplementary-Figure 4** Activation map produced by AI.

## Data Availability

The datasets analyzed during the current study are not publicly available due to limited computing/storage resources but are available from the corresponding author on reasonable request. The source code of our approach is available at GitHub: https://github.com/csu-bme/DeepPathology-CRC.

## Abbreviations

AI: Artificial intelligence
AMU: The First Affiliated Hospital Air Force Medical University
AUC: Average area under the receiver operating characteristics curve
CNN: Convolutional neural network
CGH: Chinese PLA General Hospital
CRC: Colorectal cancer
DL: Deep learning
FFPE: Formalin-fixed paraffin-embedded
FPR: False positive rate
FUS: Fudan University Shanghai Cancer Center
GPH: Guangdong Provincial People’s Hospital
HPH: Hunan Provincial People’s Hospital
H&E: Hematoxylin and eosin
NJD: Nanjing Drum Tower Hospital
PCH: Pingkuang Collaborative Hospital
SWH: Southwest Hospital
SYU: Sun Yat-Sen University Cancer Center
TXH: The Third Xiangya Hospital of CSU
WSI: Whole-slide image
XH: Xiangya Hospital
