# Supplementary material for: Accurate diagnosis of colorectal cancer based on histopathology images using artificial intelligence
Source: BMC Med. 2021 Mar 23;19:76. doi: 10.1186/s12916-021-01942-5 (PMC7986569; doi:10.1186/s12916-021-01942-5)
Supplement: Supplementary file 1 — Additional file 1: Supplementary-Text 1.a Collection and digitalization of the WSIs. Supplementary-Text 1.b Dataset-A, B, C and D. Supplementary-Text 1.c Patch-level performance and patient-level accuracy. Supplementary-Text 1.d Comparison of different architectures at patch-level. Supplementary-Text 1.e Comparison of different cluster sizes for aggregation of patch-level results. Supplementary-Table 1. Input patch size for common CNN. Supplementary-Table 2 Pathologist info. Supplementary-Table 3 List of AUCs of AI applied in CRC and other cancer types. Supplementary-Table 4 Overall performance of AI and pathologists in Human-AI contest. Supplementary-Table 5 Cohen’s Kappa coefficient for agreement among human experts and AI. Supplementary-Figure 1 Weakly-labeled and fully-labeled CRC patches. Supplementary-Figure 2.The distribution of cancerous area in multiple independent WSI datasets measured by the proportion of patches (P) containing cancer cells on the WSI. Supplementary-Figure 3 Heatmap produced by AI. Supplementary-Figure 4 Activation map produced by AI. [file 12916_2021_1942_MOESM1_ESM.docx]

**Additional File 1**

**1. Supplementary Text**

**1.a Collection and digitalization of the WSIs 2**

**1.b Dataset-A, B, C and D 3**

**1.c Patch-level performance and patient-level accuracy 4**

**1.d Comparison of different architectures at patch-level 5**

**1.e Comparison of different cluster sizes for aggregation of patch-level results 8**

**2. Supplementary Tables 10**

**3. Supplementary Figures 15**

**1.a Collection and digitalization of the WSIs**

After the data quality check introduced in the following paragraph, we collected more than 14,234 CRC WSIs from >9,185 patients from fourteen independent sources in China, U.S., and Germany (Figure S1), including: eleven hospitals (XH, TXH, HPH, PCH, FUS, AMU, NJD, GPH, CGH, SWH, SYU), a professional clinical service laboratory (ACL), and two public databases (TCGA and NCT-UMM). The number of selected patients collected on the same day was limited to less than 50 to avoid potential over-representation of WSIs prepared on that day for subsequent analyses. The personnel who completed the random selection of WSIs did not participate in subsequent research.

According to the selected pathology ID, the technicians of the pathology department obtained the slides from the pathology archive library. After simply wiping off the dust on the surface, the slides were scanned by a KF-PRO-005 scanner (KFBIO Company, Ningbo, China) at a 20× magnification. The scanning speed was ~40 seconds per slide. A quick visual examination of image quality was conducted to ensure the shape and location of tissue/cells on the digital slide was clear. Other factors, such as color differences, dirt in the background, and sharpness at the edge of cells were not considered. At this step, we removed 249 slides from 141 XH subjects and 26 slide images from TCGA that were low-resolution, unclear, obscure, or contained no tissues.


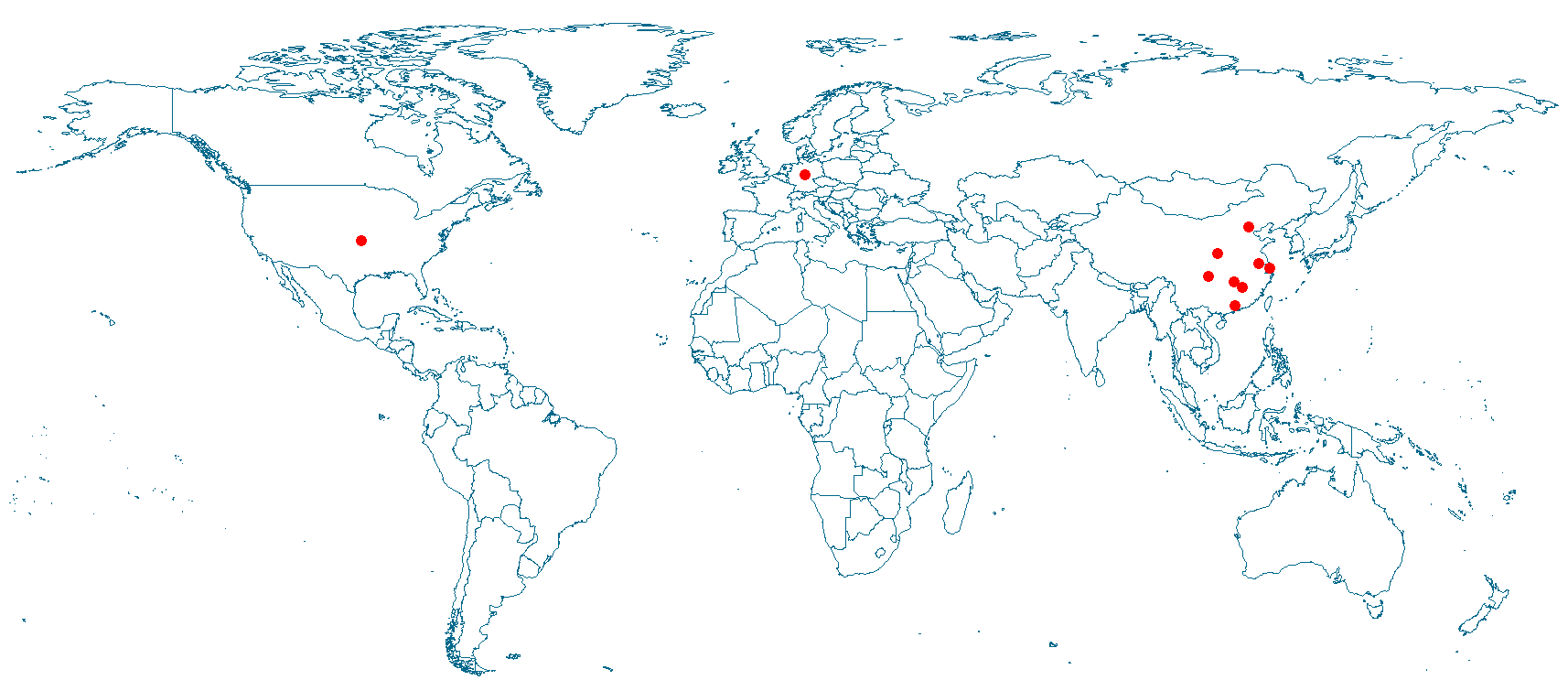
In order to better describe the nature of the images, each expert estimated the proportion of cancerous tissue in all the tissue areas. The average of their estimates was used to describe the area of the cancer tissue.

**Figure S1. Location of independent data sources indicated by red circles**

**1.b Dataset-A, B, C, and D**

The sets of images chosen from XH for Dataset-A, C, and D were mutually exclusive. We denoted them as XH-Dataset-A, XH-Dataset-C, and XH-Dataset-D accordingly. The images from other hospitals TXH, PCH, HPH, FUS, GPH, NJD, SWH, AMU, SYU, and CGH, as well as ACL were used for both patient-level testing (Dataset-C) and the Human-AI contest (Dataset-D). The Dataset-B images for patch-level validation was all from NCT-UMM. The TCGA images made from FFPE and frozen samples were used for patient-level testing only as their diagnosis is known online. The slides from different sources were distributed approximately equally in Dataset-D (Figure S2).

**
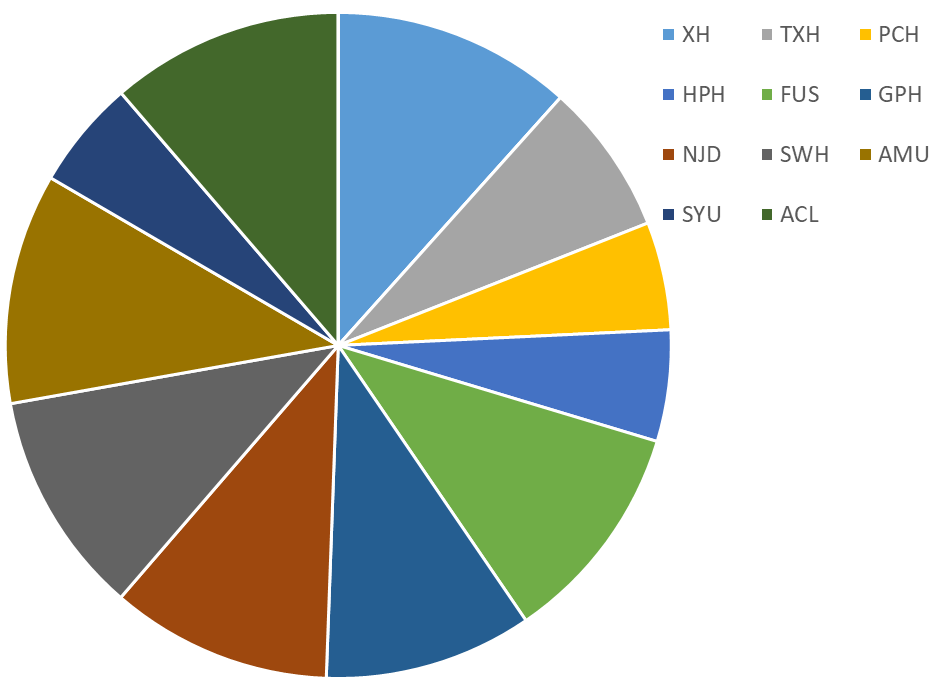
Table S1. Allocation (number) of slides in various datasets**

| **Dataset** | **A** | **B** | **C** | **D** |
| --- | --- | --- | --- | --- |
| XH | 842 | 0 | 10003 | 213 |
| NCT-UMM | 0 | 111 | 0 | 0 |
| TXH | 0 | 0 | 135 | 135 |
| PCH | 0 | 0 | 96 | 96 |
| HPH | 0 | 0 | 99 | 99 |
| FUS | 0 | 0 | 198 | 198 |
| GPH | 0 | 0 | 185 | 185 |
| NJD | 0 | 0 | 197 | 197 |
| SWH | 0 | 0 | 199 | 199 |
| AMU | 0 | 0 | 205 | 205 |
| SYU | 0 | 0 | 97 | 97 |
| ACL | 0 | 0 | 207 | 207 |
| CGH | 0 | 0 | 100 | 0 |
| TCGA | 0 | 0 | 1793 | 0 |
| **Total** | 842 | 111 | 13,514 | 1,831 |

**Figure S2. The allocation of slides in Dataset-D from different independent sources.**

**1.c Patch-level performance and patient-level accuracy**

Given the possible high false positive rate after aggregating the patch-level results, the optimal set of hyper-parameters was randomly searched with an objective of reaching >95% sensitivity and >99% specificity. We showed that, with this objective at the patch-level, the error rate at the patient-level was well controlled. We define the following metrics used in our evaluations: sensitivity (Se) = (True predicted positives)/(Real positives), specificity (Sp) = (True predicted negatives)/(Real negatives), and false positive rate (FPR) = (False predicted positives)/(Real negatives) = 1- Sp. The sensitivity measures how the model can diagnose real colorectal cancer (CRC) patients as CRC cases, while the specificity measures how the model can identify non-CRC patients as non-CRC subjects. Low sensitivity will lead to misdiagnosis and no treatment of CRC patients. Low specificity will lead to misdiagnosis and wrong treatment of non-CRC subjects.

Our approach for patient diagnosis was based on the aggregation of patch-level predictions. The performance of patch-level prediction would determine the accuracy of patient-level diagnosis. Assuming the patch-level sensitivity and specificity were $\theta$ and $\gamma$, the cluster size for aggregation was k, and each patient had only one histological image slide, if the input patches contained complete cancer or non-cancer information and were mutually independent, the theoretical probability of correctly identifying CRC patients (patient-level Se) was $\theta^{k}$, and the probability of falsely identifying non-CRC patients (patient-level FPR) was ${(1-\gamma)}^{k}$. When $k=3$, patch-level Sp=0.95, we have a patch-level FPR=0.05, while the patient-level FPR≈0.0001. Our empirical results, including examples in Table S2, showed that a patch-level sensitivity of ~95% and specificity of ~99% was sufficient to achieve a high predictive power and control the FPR at the patient-level (Supplementary-Text 1.e).

**1.d Comparison of different CNN architectures at patch- and patient-level**

In choosing the architecture for our Convolutional Neural Network (CNN), we tested and compared the relative performances in our datasets several architectures that are commonly used in histological and other image analyses, including: VGGNet [36], Inception-v1 [34], Inception-v3[26], and Inception-v4 (ResNet-v2)[35]. We compared their relative performances at patch-level (XH-Dataset-A, NCT-CRC-HE-100K in Dataset-B), patient-level (XH-Dataset-C, PCH, TXH, HPH, FUS, GPH, SWH, SYU-CGH, AMU, NJD, ACL, TCGA), and using Human-AI contest testing datasets (XH-Dataset-D, PCH, TXH, HPH, ACL). A cluster size of 4 was used for the patient-level comparison.


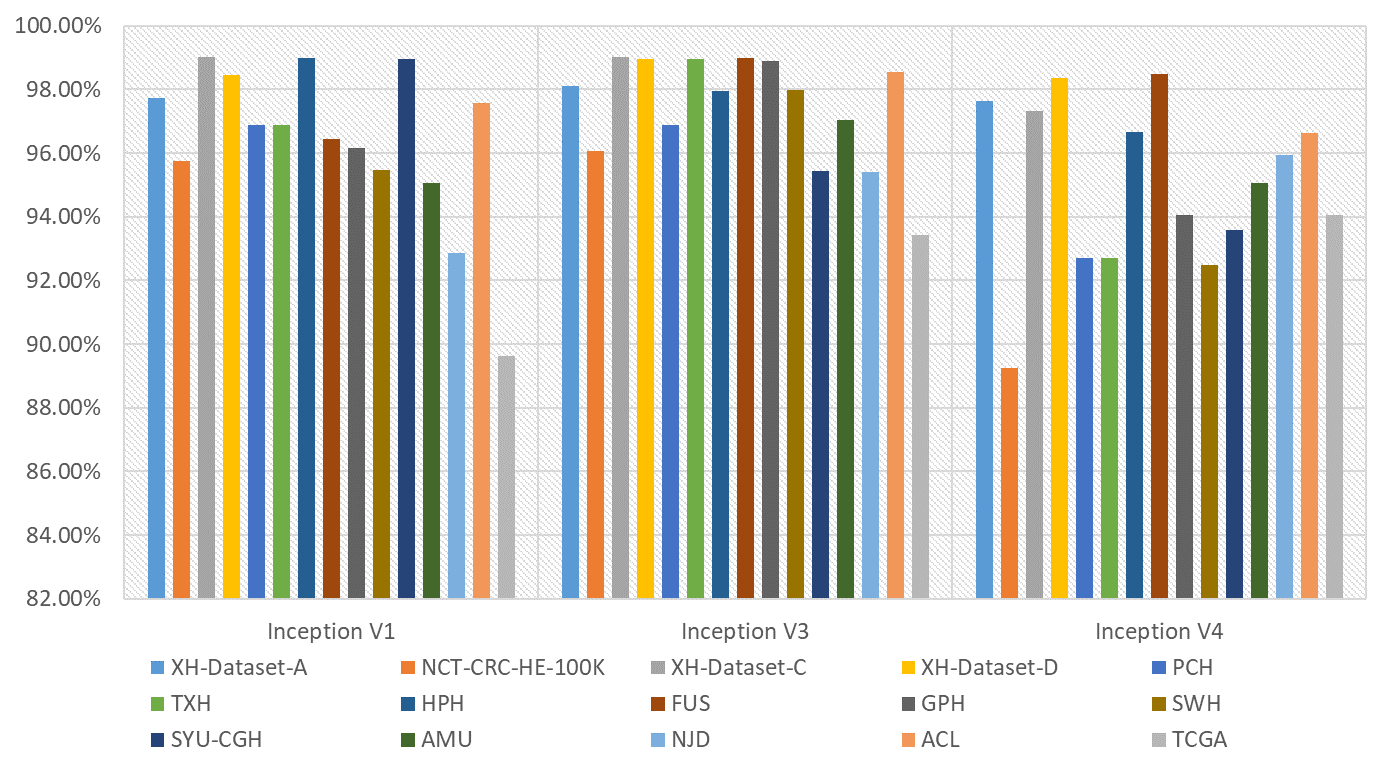
Inception-v3 was finally selected to be the architecture used in our approach. In Table S2, Inception-v3 yielded the highest accuracy and AUC using patch-level XH-Dataset-A and patient-level XH-Dataset-B, which was the largest dataset by sample size. In Figure S3, the accuracies of the Inception-v3 network were high and stable for almost all the datasets relative to Inception-v1 and Inception-v4. The only performance deficiency of Inception-v3 was on the TCGA-Frozen dataset, which may be explained by the different preparation with frozen samples and/or other sample preprocessing procedures that are different from the data sets used for training. The result from VGGNet was worse than the three Inception CNN architectures and thus not shown here. Therefore, we choose Inception-V3 as the architecture of the CNN model in our approach.

**Figure S3. Accuracies of Inception architectures on the datasets**

During the preparation of this manuscript, we are aware of that several most recent CNN architectures have been proposed after the completion of the study of the present paper, such as DenseNet[37], Squeeze-and-Excitation network[38], ResNeXt[39], etc. We did some brief analysis to compare the ResNet152V2, DenseNet201, and NASNetLarge relative to Inception-v3 in classifying CRC and non-CRC patches. Inception-v3 still performed best. DenseNet201 produced similar evaluation metrics as Inception-v3, while the other two architectures yielded less accuracy and AUC than Inception-v3.

There are two major reasons that may explain why Inception-v3 has better results in our study. First, the depth of Inception-v3 is 159 and it has ~21.8 million (M) parameters. Comparing to other architectures, Inception-v3 is deep enough with an efficient number of parameters to process the colon WSIs. For example, the depth of VGGNet is only 23-26 but there are ~138 M parameters. The depth of Inception-v1 is 22 with ~5M parameters. Second, the multi-scale modules in Inception-v3 may be more appropriate to learn the pathology in WSI relative to residual block and dense connection used in other architectures, such as the Inception-v4.

**Table S2. Relative performance of Google Inception architectures on the datasets**

| Architecture | Data source | Sensitivity | Specificity | Accuracy | AUC |
| --- | --- | --- | --- | --- | --- |
| Inception V1 | XH-Dataset-A | 95.33% | 99.31% | 97.72% | 99.77% |
|  | NCT-CRC-HE-100K | 94.31% | 95.98% | 95.74% | 98.66% |
|  | XH-Dataset-C | 98.80% | 99.51% | 99.02% | 99.16% |
|  | XH-Dataset-D | 97.96% | 98.97% | 98.46% | 98.46% |
|  | PCH | 100% | 93.48% | 96.88% | 96.74% |
|  | TXH | 98.00% | 95.74% | 96.88% | 96.85% |
|  | HPH | 100% | 97.96% | 98.98% | 98.98% |
|  | FUS | 100% | 92.86% | 96.46% | 97.96% |
|  | GPH | 100% | 91.76% | 96.17% | 96.47% |
|  | SWH | 98.99% | 92.00% | 95.48% | 98.43% |
|  | SYU-CGH | 100.00% | 83.33% | 98.97% | 91.67% |
|  | AMU | 100% | 90.20% | 95.07% | 97.55% |
|  | NJD | 95.96% | 89.69% | 92.86% | 95.71% |
|  | ACL | 99% | 96.26% | 97.58% | 97.63% |
|  | TCGA-Frozen | 89.49% | 91.04% | 89.64% | 95.45% |
| Inception V3 | XH-Dataset-A | 96.99% | 99.22% | 98.11% | 99.83% |
|  | NCT-CRC-HE-100K | 92.03% | 96.74% | 96.07% | 98.32% |
|  | XH-Dataset-C | 98.80% | 99.51% | 99.02% | 99.16% |
|  | XH-Dataset-D | 97.96% | 100.00% | 98.97% | 98.98% |
|  | PCH | 96.00% | 97.83% | 96.88% | 96.91% |
|  | TXH | 100.00% | 97.92% | 98.96% | 98.96% |
|  | HPH | 97.96% | 97.96% | 97.96% | 97.96% |
|  | FUS | 100% | 97.96% | 98.99% | 98.98% |
|  | GPH | 100% | 97.65% | 98.91% | 98.82% |
|  | SWH | 98.99% | 97.00% | 97.99% | 97.99% |
|  | SYU-CGH | 98.90% | 92.45% | 95.43% | 95.68% |
|  | AMU | 97% | 97.06% | 97.04% | 97.04% |
|  | NJD | 92.93% | 97.94% | 95.41% | 95.43% |
|  | ACL | 100% | 97.20% | 98.55% | 98.60% |
|  | TCGA-Frozen | 94.04% | 88.06% | 93.44% | 91.05% |
| Inception V4 | XH-Dataset-A | 96.95% | 98.34% | 97.64% | 97.81% |
|  | NCT-CRC-HE-100K | 94.08% | 88.42% | 89.24% | 97.10% |
|  | XH-Dataset-C | 99.33% | 96.97% | 97.32% | 98.14% |
|  | XH-Dataset-D | 97.67% | 98.97% | 98.36% | 98.32% |
|  | PCH | 98.00% | 86.96% | 92.71% | 92.48% |
|  | TXH | 100.00% | 85.42% | 92.71% | 92.71% |
|  | HPH | 100.00% | 93.33% | 96.67% | 96.67% |
|  | FUS | 100% | 96.94% | 98.48% | 98.50% |
|  | GPH | 100% | 87.06% | 94.05% | 95.70% |
|  | SWH | 100% | 85.00% | 92.50% | 93.20% |
|  | SYU-CGH | 100% | 87.46% | 93.58% | 94.10% |
|  | AMU | 100% | 90.20% | 95.07% | 96.50% |
|  | NJD | 99.00% | 92.78% | 95.93% | 95.90% |
|  | ACL | 96.00% | 97.20% | 96.62% | 96.60% |
|  | TCGA-Frozen | 93.66% | 96.36% | 94.06% | 95.01% |

**1.e Comparison of different cluster sizes for aggregation of patch-level results for patient level diagnosis**

We compared the performance of different cluster sizes on aggregating the patch-level results to patient-level prediction. The patient-level testing datasets were used for the comparison, including XH-Dataset-C, XH-Dataset-D, PCH, TXH, HPH, FUS, GPH, NJD, SWH, AMU, SYU, CGH, ACL, and frozen TCGA samples.

We selected the cluster size of 4 in our approach because it resulted in the highest accuracy in most of the datasets (except the TCGA), which also represented the best balance between sensitivity and specificity. Using the results from XH-Dataset-C as an illustrating example, the cluster size of four was able to achieve high sensitivity (98.80%) and specificity (99.51%), while the size of three yielded slightly higher sensitivity (99.52%) and lower specificity (97.78%). The cluster size of 2 was good with respect to the diagnosis rate of CRC (sensitivity 99.72%). Generally, increasing the cluster size would lead to higher specificity but lower sensitivity, which was illustrated in Figure S4 using the largest test set of XH-Dataset-C.

**Table S4. Comparison of cluster size for aggregation of patch-level results**

| Cluster Size | Data source | Sensitivity | Specificity | Accuracy | AUC |
| --- | --- | --- | --- | --- | --- |
| 2 continuous patches | XH-Dataset-C | 99.72% | 93.67% | 97.81% | 96.70% |
|  | XH-Dataset-D | 100% | 87.63% | 93.85% | 93.81% |
|  | PCH | 100% | 71.74% | 86.46% | 85.87% |
|  | TXH | 100% | 72.92% | 86.46% | 86.46% |
|  | HPH | 100% | 73.47% | 86.73% | 86.73% |
|  | FUS | 100% | 66.33% | 83.33% | 83.16% |
|  | GPH | 100% | 82.35% | 91.80% | 91.18% |
|  | NJD | 98.99% | 62.89% | 81.12% | 80.94% |
|  | SWH | 100% | 61.00% | 80.40% | 80.50% |
|  | AMU | 100% | 77.45% | 88.67% | 88.73% |
|  | SYU-CGH | 100% | 60.38% | 78.68% | 80.19% |
|  | ACL | 100% | 74.77% | 86.96% | 87.38% |
|  | TCGA-Frozen | 98.43% | 55.22% | 94.11% | 76.83% |
| 3 continuous patches | XH-Dataset-C | 99.52% | 97.78% | 98.97% | 98.65% |
|  | XH-Dataset-D | 98.98% | 93.81% | 96.41% | 96.40% |
|  | PCH | 100% | 91.30% | 95.83% | 95.65% |
|  | TXH | 100% | 83.33% | 91.67% | 91.67% |
|  | HPH | 100% | 87.76% | 93.88% | 93.88% |
|  | FUS | 100% | 90% | 94.95% | 94.90% |
|  | GPH | 100% | 92.94% | 96.72% | 96.47% |
|  | NJD | 95.96% | 85.57% | 90.82% | 90.76% |
|  | SWH | 100% | 84.00% | 91.96% | 92.00% |
|  | AMU | 99% | 89.22% | 94.09% | 94.11% |
|  | SYU-CGH | 100% | 81.13% | 89.85% | 90.57% |
|  | ACL | 100% | 89.72% | 94.69% | 94.86% |
|  | TCGA-Frozen | 97.19% | 82.84% | 95.75% | 90.01% |
| 4 continuous patches | XH-Dataset-C | 98.80% | 99.51% | 99.02% | 99.16% |
|  | XH-Dataset-D | 97.96% | 100.00% | 98.97% | 98.98% |
|  | PCH | 96.00% | 97.83% | 96.88% | 96.91% |
|  | TXH | 100.00% | 97.92% | 98.96% | 98.96% |
|  | HPH | 97.96% | 97.96% | 97.96% | 97.96% |
|  | FUS | 100% | 97.96% | 98.99% | 98.98% |
|  | GPH | 100% | 97.65% | 98.91% | 98.82% |
|  | NJD | 92.93% | 97.94% | 95.41% | 95.43% |
|  | SWH | 98.99% | 97.00% | 97.99% | 97.99% |
|  | AMU | 97% | 97.06% | 97.04% | 97.04% |
|  | SYU-CGH | 98.90% | 92.45% | 95.43% | 95.68% |
|  | ACL | 100% | 97.20% | 98.55% | 98.60% |
|  | TCGA-Frozen | 94.04% | 88.06% | 93.44% | 91.05% |


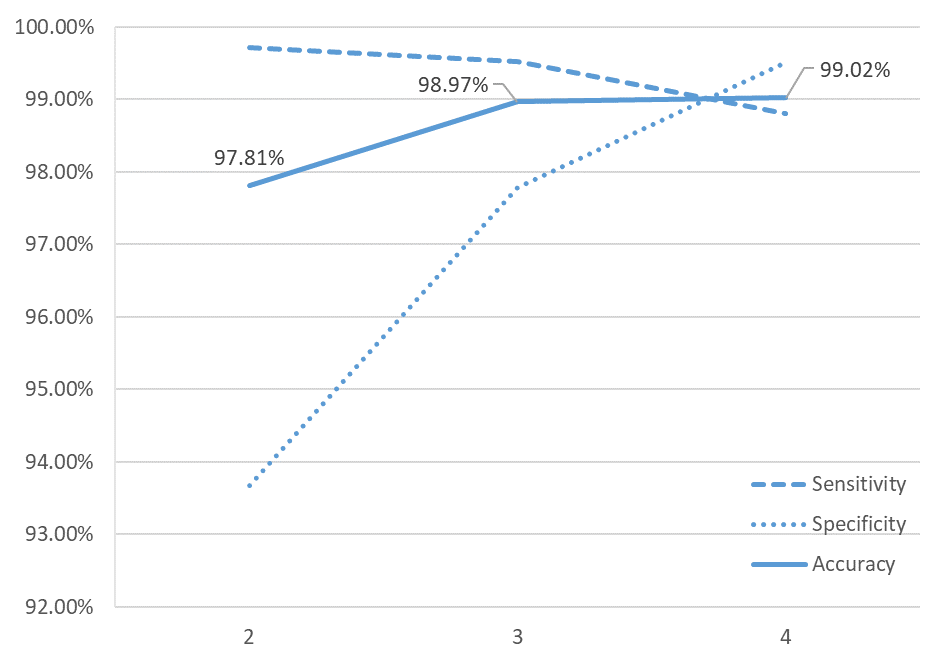
**Figure S4. Performance change with the increase of the cluster size (x-axis) on XH-Dataset-C**

**Supplementary-Table 1. Input patch size for common CNN**

| **Model and input** | **Patch size** | **Notes** |
| --- | --- | --- |
| Patches in training sets | 300*300*3 | The size of Labeled patch by Pathologists |
| Inception V1 | 224*224*3 | Default input size |
| Inception V3 | 299*299*3 | Default input size |
| Inception V4 | 299*299*3 | Default input size |
| VGG 19 | 224*224*3 | Default input size |
| ResNet-101 | 224*224*3 | Default input size |

**Supplementary-Table 2. Pathologist info**

| **Pathologist ID** | **Years in Clinic** | **Title** |
| --- | --- | --- |
| A | 1 | Resident physician |
| B | 3 | Resident physician |
| C | 5 | Physician-in-charge |
| D | 7 | Physician-in-charge |
| E | 12 | Physician-in-charge |
| F | 18 | Associate chief physician |

**Supplementary-Table** 3**. List of AUCs of AI applied in CRC and other cancer types**

| Study | Patch-level test data | | Independent patch-level test data | | Slide-level test data | | Independent slide-level test data | | |
| --- | --- | --- | --- | --- | --- | --- | --- | --- | --- |
|  | Number (#) of patches | AUC | # of patches | AUC | # of slides | AUC | # of datasets | # of slides | AUC |
| **Colorectal cancer** | | | | | | | | | |
| Haj‑Hassan et al.[19] | NA | Unsegmented~0.7923  Segmented~0.9917 | NA | NA | NA | NA | NA | NA | NA |
| Xu et al.[43] | 717 | 0.969-0.980^a^ | NA | NA | NA | NA | NA | NA | NA |
| Sari et al.[17] | 1,592 | 0.994 | NA | NA | NA | NA | NA | NA | NA |
| Kainz et al.[44] | 60 | 0.983^a^ | 20^a^ | 0.950^a^ | NA | NA | NA | NA | NA |
| Kather et al.[22] | 100,000 | 0.987 | 7,180 | 0.943 | NA | NA | NA | NA | NA |
| Ponzio et al.[45] | 4500 | 0.9037-0.9682 | NA | NA | NA | NA | NA | NA | NA |
| **Other cancers** | | | | | | | | | |
| Coudray et al.[8] | NA | NA | NA | NA | 244 | 0.990-0.993 | 3 | 340 | LUAD~0.833-0.913  LUSC~0.861-0.941 |
| Cruz-Roa et al.[46] | 50,963 | 0.842^b^ | NA | NA | NA | NA | NA | NA | NA |
| Araujo et al.[47] | 240 | 0.829^a^ | 192 | 0.693^a^ | 20 | 0.900^a^ | 1 | 16 | 0.750^a^ |
| Motlagh et al.[48] | 2,147 | 0.999 | NA | NA | NA | NA | NA | NA | NA |
| Campanella et al.[12] | NA | NA | NA | NA | 12,132 | 0.986-0.991 | 1 | 12,727 | 0.986-0.991 |
| Campanella et al.[12] | NA | NA | NA | NA | 6,252 | 0.986-0.988 | 1 | 3,710 | 0.986-0.988 |
| Campanella et al.[12] | NA | NA | NA | NA | 8,670 | 0.965-0.966 | 1 | 1,224 | 0.965-0.966 |
|  | | | | | | | | | |
| **Our study** | 20,264 | 0.998 | 107,180 | 0.983-0.985 | 10,003 | 0.992 | 12 | 3,065 | 0.911-0.992^c^ |

Note: a: accuracy; b: balanced accuracy; c, aggregated on 4 continuous patches

**Supplementary-Table 4. Overall performance of AI and pathologists in Human-AI contest**

|  | AI | Pathologists | | | | | | |
| --- | --- | --- | --- | --- | --- | --- | --- | --- |
|  |  | A | B | C | D | E | F | Average |
| Sensitivity | 98.16% | 98.08% | 97.26% | 96.71% | 98.26% | 98.53% | 96.00% | 97.47% |
| Specificity | 98.05% | 92.19% | 94.87% | 98.90% | 99.09% | 98.17% | 95.26% | 96.41% |
| Accuracy | 98.06% | 95.81% | 96.73% | 97.70% | 98.56% | 98.38% | 95.64% | 97.14% |
| AUC | 98.83% | 95.14% | 96.07% | 97.83% | 98.67% | 98.35% | 95.63% | 96.95% |

**Supplementary-Table 5. Cohen's Kappa coefficient for agreement among human experts and AI**

|  | Human Experts | | | | | |
| --- | --- | --- | --- | --- | --- | --- |
|  | A | B | C | D | E | F |
| AI | 0.891 | 0.896 | 0.905 | 0.919 | 0.908 | 0.858 |
| A |  | 0.931 | 0.924 | 0.920 | 0.920 | 0.813 |
| B |  |  | 0.938 | 0.935 | 0.921 | 0.841 |
| C |  |  |  | 0.944 | 0.928 | 0.851 |
| D |  |  |  |  | 0.945 | 0.880 |
| E |  |  |  |  |  | 0.851 |

**
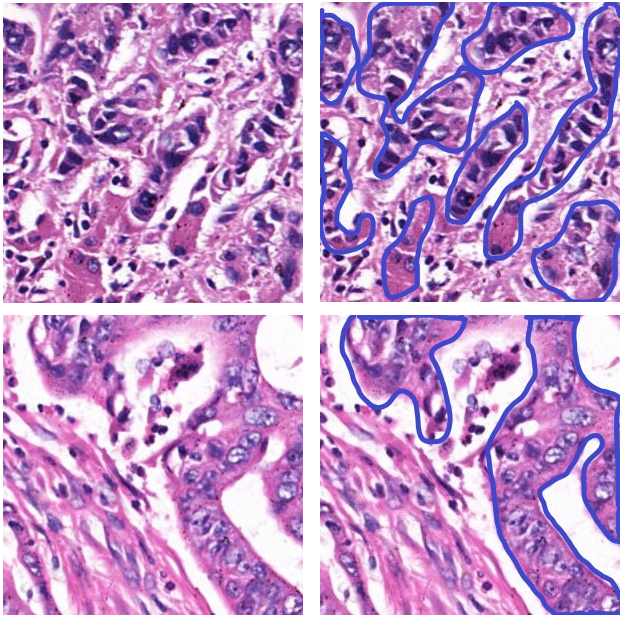
Supplementary-Figure 1. Weakly-labeled and fully-labeled CRC patches. Left column – weakly-labeled patches only labeled as cancer positive patches, right column – fully-labeled patches with the position and region of cancer cells labeled to distinguish cancel cells from the surrounding normal tissue cells.**

| P | PCH | TXH | HPH | GPH | AMU | NJD | FUS | SWH | SYU | ACL | XH-Dataset-D | XH-Dataset-C |
| --- | --- | --- | --- | --- | --- | --- | --- | --- | --- | --- | --- | --- |
| 0-1% | 4% | 0% | 7% | 0% | 3% | 1% | 0% | 1% | 7% | 0% | 1% | 10% |
| 1%-5% | 10% | 1% | 3% | 1% | 6% | 4% | 2% | 0% | 2% | 1% | 2% | 5% |
| 5%-10% | 10% | 4% | 3% | 0% | 3% | 8% | 5% | 4% | 1% | 2% | 2% | 7% |
| 10%-20% | 21% | 25% | 14% | 3% | 14% | 12% | 8% | 5% | 6% | 12% | 19% | 19% |
| 20%-30% | 19% | 32% | 26% | 13% | 17% | 22% | 23% | 9% | 18% | 19% | 16% | 22% |
| 30%-50% | 29% | 35% | 38% | 43% | 38% | 40% | 53% | 52% | 40% | 40% | 37% | 31% |
| 50%-100% | 7% | 3% | 9% | 40% | 19% | 13% | 9% | 29% | 26% | 26% | 23% | 6% |

**Supplementary-Figure 2. The distribution of cancerous area in multiple independent WSI datasets measured by the proportion of patches (P) containing cancer cells on the WSI. The estimate of proportions was provided by the pathologist.**

******Supplementary-Figure 3. Heatmap produced by AI. Left column – WSI, middle column – predicted heatmap, right column – heatmap overlaid on the WSI. Top row: positive case from radical surgery; second row: negative case from radical surgery; third row: positive case colonoscopy; bottom row: negative case colonoscopy.**

**Supplementary-Figure 4. Activation map produced by AI. Left column – WSI, right column – activation map. Each row is a pair of WSI and activation map from the same patch. The heat color indicates informative regions used by the DL AI for CRC detection.**
